# 

**Published:** 1877-02

**Authors:** 


### CONTENTS:

ORIGINAL COMMUNICATIONS:

A Case of Uterine Fibro-Cystoma. By Franklin Staples, M.D. .. 97

Atrophiæ Sulphas in Acute Myringitis and Otitis. By A. N. Ellis, M.D. .......... 104

The Silk Worm Epidemic, Pebrine, after Pasteur, with some reflections Regarding the Analogy of this Disorder to Certain Diseases of the Human Subject. By John Bartlett, M.D. ..... 108

The Mortality of Surgical Operations in the Upper Lake States, Compared with that of Other Regions. By E. Andrews, M.D.. 121

CORRESPONDENCE:

Solvent for Salicylic Acid .......... 131

The Corn Sweat .......... 132

BOOK REVIEWS .......... 133

BOOKS AND PAMPHLETS RECEIVED .......... 147

CLINICAL REPORTS .......... 148

MEDICAL NEWS AND ITEMS .......... 153

EDITORIAL .......... 160

OBITUARY .......... 161

SUMMARY OF PROGRESS IN THE MEDICAL SCIENCES:

I. Practical Medicine .......... 162

II. Surgery .......... 175

III. Obstetrics and Gynæcology .......... 183

IV. Therapeutics .......... 185

V. Ophthalmology .......... 188

VI. Syphilis and Venereal Diseases .......... 190

ANNOUNCEMENTS FOR THE MONTH .......... 192

